# Supplementary material for: Causal Relationship between Aging and Anorexia Nervosa: A White-Matter-Microstructure-Mediated Mendelian Randomization Analysis
Source: Biomedicines. 2024 Aug 16;12(8):1874. doi: 10.3390/biomedicines12081874 (PMC11351342; doi:10.3390/biomedicines12081874)
Supplement: Supplementary file 1 [file biomedicines-12-01874-s001.zip › Supplementary Figure/Figure S4/Singe SNP plot of MR analysis for TL to ubm-b-1891.pdf]

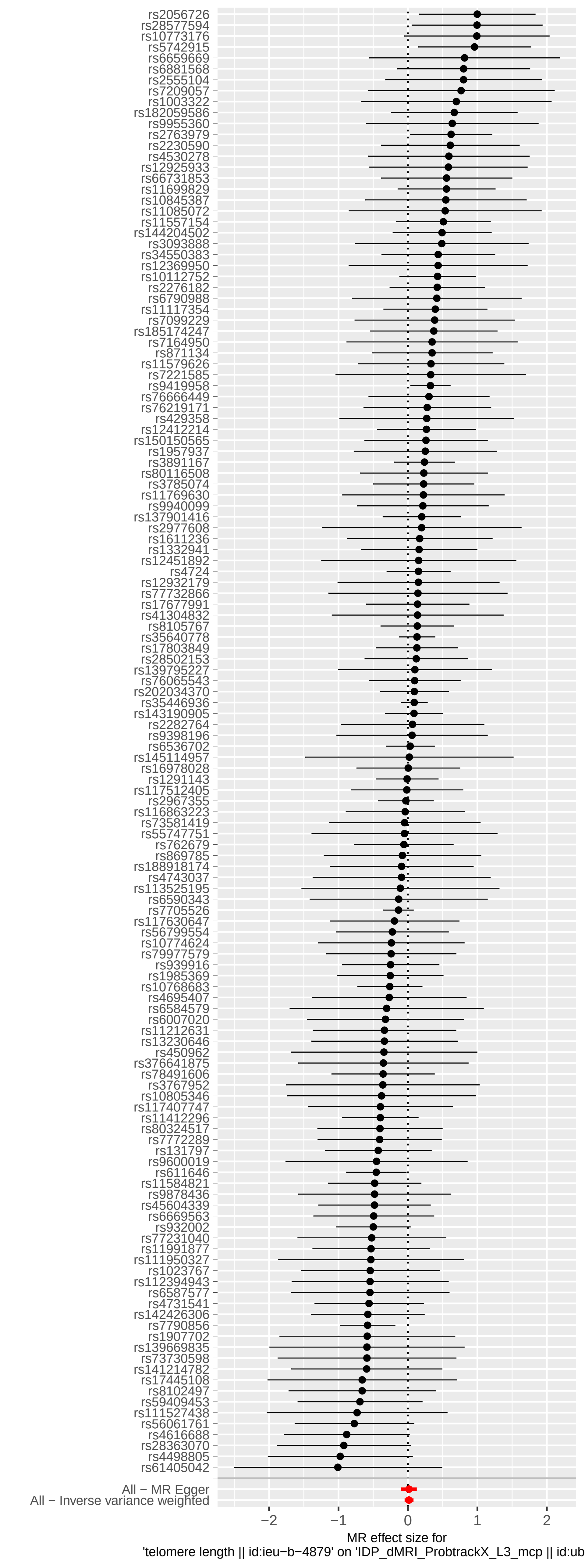

rs2056726
rs28577594
rs10773176
rs5742915
rs6659669
rs6881568
rs2555104
rs7209057
rs1003322
rs182059586
rs9955360
rs2763979
rs2230590
rs4530278
rs12925933
rs66731853
rs11699829
rs10845387
rs11085072
rs11557154
rs144204502
rs3093888
rs34550383
rs12369950
rs10112752
rs2276182
rs6790988
rs11117354
rs7099229
rs185174247
rs7164950
rs871134
rs11579626
rs7221585
rs9419958
rs76666449
rs76219171
rs429358
rs12412214
rs150150565
rs1957937
rs3891167
rs80116508
rs3785074
rs11769630
rs9940099
rs137901416
rs2977608
rs1611236
rs1332941
rs12451892
rs4724
rs12932179
rs77732866
rs17677991
rs41304832
rs8105767
rs35640778
rs17803849
rs28502153
rs139795227
rs76065543
rs202034370
rs35446936
rs143190905
rs2282764
rs9398196
rs6536702
rs145114957
rs16978028
rs1291143
rs117512405
rs2967355
rs116863223
rs73581419
rs55747751
rs762679
rs869785
rs188918174
rs4743037
rs113525195
rs6590343
rs7705526
rs117630647
rs56799554
rs10774624
rs79977579
rs939916
rs1985369
rs10768683
rs4695407
rs6584579
rs6007020
rs11212631
rs13230646
rs450962
rs376641875
rs78491606
rs3767952
rs10805346
rs117407747
rs11412296
rs80324517
rs7772289
rs131797
rs9600019
rs611646
rs11584821
rs9878436
rs45604339
rs6669563
rs932002
rs77231040
rs11991877
rs111950327
rs1023767
rs112394943
rs6587577
rs4731541
rs142426306
rs7790856
rs1907702
rs139669835
rs73730598
rs141214782
rs17445108
rs8102497
rs59409453
rs111527438
rs56061761
rs4616688
rs28363070
rs4498805
rs61405042
All − MR Egger
All − Inverse variance weighted
−2
−1
0
1
2
MR effect size for
'telomere length || id:ieu-b-4879' on 'IDP_dMRI_ProbtrackX_L3_mcp || id:ub
